# Anxiolytic-*like* effect of *Urena lobata* (L.) in *swiss* albino mice

**DOI:** 10.1186/s40816-021-00249-5

**Published:** 2021-01-20

**Authors:** Muhammad Torequl Islam, Thoufiqul Alam Riaz, Seyed Abdulmajid Ayatollahi, Javad Sharifi-Rad

**Affiliations:** 1 Department of Pharmacy, Life Science Faculty, Bangabandhu Sheikh Mujibur Rahman Science and Technology University, Gopalganj (Dhaka), 8100 Bangladesh; 2 Department of Pharmacology, School of Medicine, Institute of New Drug Development, Jeonbuk National University, Jeonju, 54907 South Korea; 3 Phytochemistry Research Center, Shahid Beheshti University of Medical Sciences, Tehran, Iran; 4 Department of Pharmacognosy and Biotechnology, School of Pharmacy, Shahid Beheshti University of Medical Sciences, Tehran, Iran; 5 Facultad de Medicina, Universidad del Azuay, Cuenca, Ecuador

**Keywords:** *Urena lobata*, Anxiety, *Mus musculus*, Behavioral study

## Abstract

Anxiety disorders are general and psychological problems that are also linked to symptoms of depression. This study aimed to investigate the anxiolytic-*like* effects of *Urena lobata* L. (MEUL) methanolic extract in different behavioral paradigms in *Swiss* albino mice. For this, after an oral acute toxicity study, adult male mice were treated with MEUL (250 and 500 mg/kg, p.o.) and/or diazepam (2 mg/kg, i.p.), and subjected to a number of behavioral studies. In the open-field test, the number of square field cross, grooming, and rearing, was counted, while in the light/dark and swing test, the time spent in the dark portion and number of swings was calculated, respectively. Additionally, the phytochemical analysis was also done. Results reveal that the MEUL possesses alkaloids, glycosides, flavonoids, phenols, saponins, terpenes (including triterpenes), gums, and reducing sugars. MEUL showed a significant (*p* < 0.05) anxiolytic-*like* effect in experimental animals, where it’s dose-dependently modulated the test parameters in an open-field test. The MEUL also increased the light residence time and the number of swings in a dose-dependent manner. A dose of 500 mg/kg of MEUL caused the highest calming effect when combined with the experimental animals’ diazepam group. Taken together, findings expand an understanding of the impact of *U. lobata* on the central nervous system and show that this plant may be useful for the treatment of disorders associated with anxiety.

## Introduction

Herbal medicines (also called green medicinal products) are relatively healthy and effective paradigms of health care. In the last few years, traditional herbal medicine has drawn researchers' attention to the world because of its several pharmacological activities, economic viability, and fewer side effects [6]. The use of herbal medicine to treat many psychiatric disorders has become more established, as traditional pharmacotherapy is considered safer support [32].

Compare with other disease areas, developing a new drug for the central nervous system (CNS) is a unique challenge [7]. Drugs acting on the CNS, such as CNS depressants including barbiturates and benzodiazepines (BDZs), among others, exert their effect through interaction with the postsynaptic gamma-aminobutyric acid (GABA) receptor [13]. These drugs are not so safe, and they are toxic. For example, benzodiazepines and Z-drugs (e.g., zopiclone, zolpidem) are evident to cause cognitive problems at low doses [34]. These drugs also build psychological and physiological dependence in patients [9, 16]. BDZs are widely used as CNS depressants, which have been reported to develop tolerance and physical dependence [12, 33], making us think about new safer CNS depressants. Drugs from natural sources may be a good solution; for example, Luvah and coworkers introduced *Ajuga remota* (Lamiaceae) components with anxiolytic-*like* effects [21].

Similarly, Schier and colleagues' research suggested that cannabidiol (CBD) exhibits an anti-anxiety and anti-depressant effects in animal models. Also, experiments with CBD indicated non-activation of neuroreceptors CB1 and CB2 and demonstrated a good interaction with the 5-hydroxytryptamine-1A (5-HT1A) neuro-receptor [29]. However, recent findings have clearly shown that CBD is an effective drug for Panic Disorder (PD) treatment, both in animal models and in healthy volunteers. Therefore, it is a need for further research on determining the mechanism for CBD’s action and the safe and optimal doses of the compound [26].

*Urena lobata* L. (Family: Malvaceae), a shrubby herb widely distributed annually throughout the world, including Africa, Asia, and South America [4]. *U. lobata* is commonly used in the treatment of diabetes, gonorrhea, malaria, dysentery, abdominal colic, nausea, rheumatism, and edema in folk medicines [27, 31]. Scientific reports indicate that the plant has anti-bacterial, anti-hyperglycemic, anti-nociceptive, anti-diarrheal, anti-inflammatory, and wound healing activities [8, 22, 28]. A recent study suggests that compounds from *U. lobata* can inhibit nitric oxide production in lipopolysaccharide-induced RAW264.7 macrophage cells [20]. In China, *U. lobata* is clinically used to treat pathological leucorrhea and gonorrhea [23]. To date, a number of bioactive constituents have been isolated from this medicinal herb; these include flavonoids, phenylethyl glycosides, lignans, coumarins, triglycerides [14, 20, 25, 35, 37], megastigmane glycosides [36], urenalignosides A–D, and lignins [20].

This study aims to examine the anxiolytic-*like* effect of the methanol bark aerial parts extract of *U. lobata* in *Swiss* mice.

## Materials and methods

### Collection, identification, and extraction of plant materials

The aerial parts of *U. lobata* were obtained during December from the Chittagong Hill Tracts. Taxonomist, Bangladesh Forest Research Institute (BFRI), Chittagong, Bangladesh, identified the plant. There has been a collection of valuables (BFRIH-7011).

The plant materials were dried (temperature not exceeding 45 ^○^C) and made into a coarse powder using a suitable grinder. Approximately 250 gm powdered materials were soaked into 1.2 L absolute methanol with occasionally manual stirring for 14 days. Then the soaked materials were filtered through a surgical cotton plug and followed by filtration through Whatman filter paper No. 1 grade. The solvent was evaporated by using a rotary evaporator. The yield value was 9.11%.

### Drugs and chemicals

Diazepam (DZP, Square Pharmaceuticals Ltd.) was used as a standard drug. Tween 80 and all the other reagents and chemicals were purchased from Sigma-Aldrich, Denmark.

### Phytochemical investigations

The methanol extract of the plant was screened for various phytoconstituents according to the procedure described by Khandelwal [18].

### Experimental animals

*Swiss* albino male mice (22-28 g) were obtained from the animal house facilities of the Bangladesh Council for Scientific & Industrial Research (BCSIR), Chittagong, Bangladesh. The animals were free to access food and water *ad libitum* and were housed in polypropylene cages at a temperature (25 ±1 °C) throughout the study. The experiments were conducted from 9.00 am to 2.00 pm. After the examination, all animals were euthanized with sodium pentobarbital (135 mg/kg, i.p.) and were sacrificed. The experimental protocol was approved (#SUB-TI-14) by the Department of Pharmacy, Southern University Bangladesh (SUB), Chittagong, Bangladesh.

### Acute toxicity study

We followed the OECD guidelines for this test. Albino mice (n = 6) were randomly selected in this study. The animals fasted only with free access to the water for 12 hours. The animals were then weighed and orally tested at 1000, 2000, 3000, and 4000 mg/kg for extract. Then the animals had been deprived of their food for 2 hours and the first four hours afterward for 72 hours, and afterward for 7 days after administration of the drugs, mortality and clinical signs (e.g., skin, fur, eyes, and mucosal signs) were noted. Gross conduct such as body position, locomotion, raising, tremors, and gait have been observed for seven days. The impact of plant extracts on the grip strength, pain response, and an accurate reflection has been recorded. Therefore, food and water consumption are tracked.

## Test for anxiolytic-*like* effects

### Experimental design

To study the anxiolytic effect of MEUL, thirty-five mice were randomly divided into seven groups of five animals each (n = 5). Group I: Negative control (vehicle: 0.05% tween 80 dissolved in 0.9% NaCl solution); group II: Positive control (DZP); groups III and IV were treated with MEUL: 250 and 500 mg/kg), respectively; groups V and VI were treated with a combination of DZP2 and MEUL (DZP2 + MEUL 250 and DZP2 + MEUL 500).

## Open-field test (OFT)

This test was performed according to a published procedure with slight modification [3]. According to this procedure, animals received controls and test extract 30 min before the test. Then the animals were individually placed in the center of the box and allowed to move freely. The number of squares crossed with all four paws, rearings, and a number of grooming were recorded for 3 min (testing period). In the combined treatment groups, DZP (2 mg/kg, i.p.) was given 15 min before MEUL administration, and parameters mentioned earlier were similarly recorded.

## Light-dark test (LDT)

After 30 min of administration of controls and test samples, mice were placed in the middle of the light compartment. The animals were then observed for 3 min, and the time spent inside the dark box was recorded [15]. In the combined treatment groups, DZP (2 mg/kg, i.p.) was administered 15 min before the MEUL administration, and the above-mentioned parameter was similarly recorded.

## Swing test (SWT)

In this test, the number of swings of each animal was recorded for 3 min, after 30 min of administration of controls and MEUL [17]. In the combined treatment groups, DZP (2 mg/kg, i.p.) was given 15 min before the MEUL administration, and a number of swings were recorded similarly.

### Statistical analysis

Results are expressed as the mean ± standard error of the mean (SEM). Data were analyzed by one-way analysis of variance (ANOVA) Neuman Keul’s test using Graph Pad Prism (version: 6.0) software, considering *p*≤ 0.05 at 95% confidence intervals.

## Results

The phytochemical reports suggest that the plant contains alkaloids, glycosides, flavonoids, terpenes, saponins, gums, and reducing sugars (Table 1).

**Table 1:** Phytochemical relevance of the methanolic crude extract of *U. lobata*

Phytochemical groups	Test results
Alkaloids	**++**
Glycosides	**+++**
Flavonoids	**+++**
Terpenoids	**+++**
Steroids	**++**
Tannins	**-**
Saponins	**++**
Gums	**+**
Reducing Sugars	**+**

Intensity: (-) → No, (+) → Low, (++) → Moderate, (+++) → Strong

The MEUL (1000, 2000, 3000, and 4000 mg/kg, p.o.) did not change the frequently observed parameters (e.g., writing, grooming, corneal reflex, touch response, torch response, pain response, tremors, convulsion, righting reflux, skin color,pinna reflex, restlessness, alertness, pupils, urination, food intake, gripping, lacrimation, salivation, and water intake) in the experimental animals (Table 2). The extract did not exert toxic or lethal effects, suggesting the median lethal dose (LD_50_) of the extract might be more significant than 4000 mg/kg (p.o.).

**Table 2:** Effect of methanolic extract of *U. lobata* on acute oral toxicity test in *Swiss* albino mice

Observed parameters	Responses				
	0 mg/kg	1000 mg/kg	2000 mg/kg	3000 mg/kg	4000 mg/kg
Alertness	N	N	N	N	N
Grooming	A	A	A	A	A
Restlessness	A	A	A	A	A
Touch response	N	N	N	N	N
Torch response	N	N	N	N	N
Pain response	N	N	N	No	N
Tremors	A	A	A	A	A
Convulsion	A	A	A	A	A
Righting reflux	N	N	N	N	N
Gripping	N	N	N	N	N
Pinna reflex	P	P	P	P	P
Corneal reflex	P	P	P	P	P
Writhing	A	A	A	A	A
Pupils	N	N	N	N	N
Urination	N	N	N	N	N
Salivation	N	N	N	N	N
Skin color	N	N	N	N	N
Lacrimation	N	N	N	N	N
Food intake	N	N	N	N	N
Water intake	N	N	N	N	N
Mortality	-	-	-	-	-

*N* Normal, *A* Absent, *P* Present, (-) – Nil

Shown in Table 3 are the results of our investigation pertaining to the anxiolytic-*like* effects of the MEUL. Results reveal that DZP (2 mg/kg) significantly (*p* <0.05) reduced the number of field cross, rearing, and grooming in OFT. DZP also decreased the light residence time and number of swings in experimental animals in comparison to the NC and MEUL groups. MEUL dose-dependently exerted a calming effect in experimental animals. MEUL at 500 mg/kg significantly reduced the number of square crossing, rearing, and grooming parameters in OFT. MEUL at 500 mg/kg also significantly increased the light residence time and number of swings in *Swiss* mice.

**Table 3:** Effects of test sample and controls on mice

Treatment groups		Open-field test (3 min)			Light-dark test (3 min)	Swing test (3 min)
		***Field cross***	***Rearing***	***Grooming***	***Residence in light (Sec)***	***Number of swings***
**NC (10 mL/kg, p.o.)**		35.22 ± 0.87	7.08 ± 1.13	9.03 ± 1.25	153.13 ± 2.17	37.25 ± 2.19
**DZP (2 mg/kg, i.p.)**		17.05 ± 1.46*	3.57 ± 0.64*	4.41 ± 0.56*	56.12 ± 2.97*	9.33 ± 1.29*
**MEUL (mg/kg, p.o.)**	**250**	22.64 ± 1.75*	4.13 ± 1.28*	4.32 ± 1.64*	127.65 ± 2.87*	31.54 ± 2.63*
	**500**	19.75 ± 2.61*	4.11 ± 2.03*	4.21 ± 1.29*	153.02 ± 2.43	21.12 ± 2.43*

Values are the mean ± SEM (*n* = 5). ANOVA followed by Neuman Keuls test, *p < 0.05 when compared to NC group. *NC* Negative control (Vehicle: 0.05% tween 80 dissolved in 0.9% NaCl solution), *DZP* Diazepam, *MEUL* Methanol extract of *U. lobata*

Moreover, MEUL (250 and 500 mg/kg), co-treated with the DZP (2 mg/kg, i.p.) group, caused an anxiolytic-*like* effect through modifying the test parameters in OFT, LDT, and SWT. In this case, the MEUL at 500 mg/kg showed better activity than its 250 mg/kg counterpart (Table 4).

**Table 4:** Combined effects of the test sample and DZP in *Swiss* mice

Treatment groups	Open-field test (3 min)			Light-dark test (3 min)	Swing test (3 min)
	***Field cross***	***Rearing***	***Grooming***	***Residence in light (Sec)***	***Number of swings***
**NC (10 mL/kg, p.o.)**	35.22 ± 0.87	7.08 ± 1.13	9.03 ± 1.25	153.13 ± 2.17	37.25 ± 2.19
**DZP**	17.05 ± 1.46*	3.57 ± 0.64*	4.41 ± 0.56*	56.12 ± 2.97*	9.33 ± 1.29*
**DZP2 + MEUL250**	19.04 ± 2.03*^#^	3.39 ± 0.24*	4.01 ± 1.67*	141.61 ± 3.07*^#^	28.13 ± 1.03*^#^
**DZP2 + MEUL500**	23.05 ± 2.68*^#^	3.19 ± 1.07*^#^	3.04 ± 2.13*^#^	151.11 ± 2.39*^#^	34.01 ± 2.03*^#^

Values are the mean ± SEM (*n* = 5). ANOVA followed by NeumanKeuls test, *p < 0.05 when compared to the NC group. NC: Negative control (Vehicle: 0.05% tween 80 dissolved in 0.9% NaCl solution). ^#^*p* < 0.05 when compared to the positive control (DZP) group. *DZP* Diazepam, *MEUL* Methanol extract of *U. lobata*

## Discussion

Medicinal plants’ usage plays a crucial role in the complementary and alternative medicine system and has been acquired much interest in developed countries. Acute toxicity study followed by OECD guidelines helps us determine a safe dose of any substance (e.g., crude extracts, isolated compounds, food additives, chemical ingredients using laboratory animals (e.g., rats, mice, rabbits). The World Health Organization (WHO) reports that medicinal plants have been a unique ingredient in modern medicine since ancient times. However, it is important to know the appropriate and safe dose in a human before starting treatment with them [2]. We can confirm that using different animals in the laboratory in the method of acute oral toxicity analysis. In our study, MEUL did not show poisoning effects on the *Swiss* mice up to 4000 mg/kg oral dose; therefore, this dose may be considered for this category of the experimental animal.

Anxiety is a common issue at present, especially since the novel coronavirus disease (Covid-19) pandemic has increased its levels one step further worldwide [24, 39]. So far, several psychiatric drugs are being used to treat anxiety. Unfortunately, these drugs have many side effects. However, we can use herbal medicines to approach this disease [5, 10]. So focusing on traditional herbal medicine is a common fashion nowadays [38]. For example, compounds derived from medicinal plants have immunomodulatory effects; people have been using them safely since ancient times [19]. *U. lobata* has various important biological activities, including antioxidant and neuropharmacological effects [17]. Based on the above scientific, we have evaluated its anxiolytic effect in *Swiss* mice taking a popularly used standard anxiolytic drug DZP.

Anxiety is caused by an animal’s isolation and agoraphobia from its social community [1]. In the OFT, locomotion declines (diminished exploration), often involving anxieties, were found. In this study, MEUL was found to modulate OFT and SWT parameters, along with an increase in light residence time of experimental animals.

BDZs (e.g., DZP) are positive allosteric modulators of the GABA_A_. GABA is the most critical inhibitor of the brain that increases chloride ion conductivity through neuronal cells’ membrane following binding to BDZs. It triggers the influx of chloride ions to hyperpolarize the neurons’ membrane potential, which increases the difference between rest and threshold potential, thereby lowering nerve firing. Therefore, arousal of the cortical and limbic systems is decreased in the CNS [30]. To exert a calming effect, DZP appears to act on areas of the limbic system, thalamus, and hypothalamus. In this context, our results show that MEUL at a dose of 500 mg/kg may elicit a better calming effect on the experimental animals. This effect may be confirmed by reducing the movement of animals in the open-field and swing boxes and time spent in the light chamber of the dard-lightbox. Moreover, the MEUL was also found to modulate the calming effect of DZP in experimental animals, where the extract at both doses plus 2 mg/kg of DZP groups showed better anxiolytic-*like* effects than the individually treated groups with MEUL or DZP.

Researches have demonstrated that plant extracts containing a wide range of phytochemicals, including alkaloids, glycosides, terpenes, flavonoids, phenolic acids, lignans, cinnamates, and saponins, may exert anxiolytic-*like* effects in animal models [11]. Our findings demonstrated that MEUL contains alkaloids, glycosides, flavonoids, phenols, saponins, and terpenes.

## Conclusions

MEUL contains important plant secondary metabolites, including alkaloids, glycosides, flavonoids, and terpenes. The extract exhibited a calming effect in *Swiss* mice in a dose-dependent manner and modulated the standard drug’s anxiolytic effects, DZP. The extract was found to potentiate the anxiolytic-*like* effect of DZP. Further studies are required to isolate the plant’s responsible, active constituents and understand the possible mechanism of action behind the anxiolytic-*like* effect in experimental animals. Other studies are needed to determine the safety, effectiveness, and active components of this plant.

## Abbreviations

5-HT1A: 5-Hydroxytryptamine-1A
BDZs: Benzodiazepines
CBD: Cannabidiol
CNS: Central nervous system
DZP: Diazepam
GABA: Gamma-aminobutyric acid
MEUL: Methanolic extract of *Urena lobata*
